# An intervention program to reduce the number of hospitalizations of elderly patients in a primary care clinic

**DOI:** 10.1186/1472-6963-8-36

**Published:** 2008-02-06

**Authors:** Roni Peleg, Yan Press, Maya Asher, Tatyana Pugachev, Hadas Glicensztain, Mila Lederman, Aya Biderman

**Affiliations:** 1 Clalit Health Services, Southern District, Israel; 2 Department of Family Medicine, Faculty of the Health Sciences, Ben-Gurion University of the Negev, Beer-Sheva, Israel

## Abstract

**Background:**

The elderly population consumes a large share of medical resources in the western world. A significant portion of the expense is related to hospitalizations.

**Objectives:**

To evaluate an intervention program designed to reduce the number of hospitalization of elderly patients by a more optimal allocation of resources in primary care.

**Methods:**

A multidimensional intervention program was conducted that included the re-engineering of existing work processes with a focus on the management of patient problems, improving communication with outside agencies, and the establishment of a system to monitor quality of healthcare parameters. Data on the number of hospitalizations and their cost were compared before and after implementation of the intervention program.

**Results:**

As a result of the intervention the mean expenditure per elderly patient was reduced by 22.5%. The adjusted number of hospitalizations/1,000 declined from 15.1 to 10.7 (29.3%). The number of adjusted hospitalization days dropped from 132 to 82 (37.9%) and the mean hospitalization stay declined from 8.2 to 6.7 days (17.9%). The adjusted hospitalization cost ($/1,000 patients) dropped from $32,574 to $18,624 (42.8%). The overall clinic expense, for all age groups, dropped by 9.9%.

**Conclusion:**

Implementation of the intervention program in a single primary care clinic led to a reduction in hospitalizations for the elderly patient population and to a more optimal allocation of healthcare resources.

## Background

The rise in life expectancy in the western world has led to a significant increase in the number of individuals over the age of 65 years. The percentage of elderly has reached 19.1% in Italy, ranked the world's oldest country, followed by Japan at 19% [1].

In Israel at the end of 2002 there were approximately 655,000 elderly individuals, aged 65 and above, comprising just under 10% of the population [2]. Life expectancy in Israel is among the highest in the world and is currently 81.2 years for women and 77.3 years for men. While the elderly population increased in absolute terms by 48% in the years 1990 to 2002, the number of those older than 80 years grew by 69% [2]. This population group is particularly susceptible to cognitive and functional impairment [3-5], depression [6,7], falls [8], and visual [9] or hearing impairment [10]. The elderly population is the largest consumer of healthcare resources in the western world. A large part of these expenses stems from the hospitalization of elderly patients in medical centers. They are also more likely to have inadequate financial stability and to be unaware of available social support services [11]. Frequently, such conditions are overlooked by the primary care physician [12,13].

Improvement in the care of elderly patients and more optimal use of healthcare resources, including hospitalizations, represents an important challenge for healthcare services in the world.

In the study clinic the adjusted per patient cost is high compared to regional and national averages with an increase in costs over the years. The primary cause of this disparity is the cost of hospitalization, primarily among patients 65 years of age and above.

The objective of the present study was to evaluate the results of an intervention program designed to improve care for elderly patients, to reduce hospitalizations, and to generate a more optimal use of healthcare resources.

## Methods

### Setting

The compulsory national health insurance system, implemented in Israel in 1995, provides healthcare to the entire population through non-profit health maintenance organizations (sick funds). Four sick funds provide healthcare services to the Israeli population based on the patient-oriented medicine model, which entails the provision of treatment by a primary care physician and a nurse working as a team, and specialist consultations as required. The present study was conducted within the framework of a quality improvement program in the Clalit Health Services (CHS) for the southern district of Israel. CHS is Israel's largest sick fund, serving about 53% of the population.

The intervention program and its evaluation were conducted in a primary care clinic in Beer-Sheva, the capitol city of the southern district. The clinic, which is located about 5 minutes by foot from the central city hospital (Soroka Medical Center), provides primary healthcare services for 4,620 registered clients of all ages. The number of insured elderly patients 65 years old or above is 808, 17.5% of the clinic's registry. The clinic is located in one of the city's older neighborhoods. The population is generally of low socioeconomic status with 22.6% receiving support from Israel's Social Security Institute compared to 13.0% of the registered clients of CHS throughout the country. The clinic staff includes three family physicians and one pediatrician, two nurses, an administrative director, and a part-time administrator whose task is to promote facilitate the independent functioning of the clinic. All medical records are computerized and statistical and economic data are available, including quality of care indicators. Patients mostly have scheduled appointments with the doctors at ten minutes intervals. The clinic has an annual budget for which it is independently responsible.

### Baseline data prior to implementation of the intervention program – patients 65 years or above

During the course of 2004 the mean expenditure for patients 65 years of older was $2,273, compared to a mean of $1,840 for the region (a difference of 21.1%). The adjusted mean number of hospitalizations per 1,000 patients was 15.1 in the clinic compared to 11.1 for the region (a difference of 35.7%). The adjusted mean number of hospitalization days per 1,000 was 132 compared to 82 for the region (59%), and the adjusted mean cost of hospitalization per 1,000 in the clinic was $32,574 compared to $21,794 for the region (49.5%). The mean duration of hospitalization for the clinic was 8.2 days compared to 7.2 for the region. Data on hospitalizations for elderly patients appear in Table 1.

**Table 1:** Hospitalizations for patients 65 and older in 2004 (1 USD = 4.45 NIS).

	Southern district	Study clinic	Difference (%)
Cost per registered patient ($)	1,840	2,227	387 (21.1)
Adjusted hospitalizations/1,000 patients	11.1	15.1	3.97 (35.7)
Adjusted duration of hospitalization (days/1,000)	83	132	49 (59)
Adjusted hospitalization cost ($/1,000)	21,794	32,574	10,780 (49.5)
Mean duration of hospitalization (days)	7.2	8.2	1 (13.8)

### Definition of the problem

There is a significantly higher cost of hospitalization for elderly patients between the study clinic and the regional average.

### Analysis of the causes of this problem

As a first step the clinic staff analyzed the causes of the high costs of hospitalization.

#### The medical system problem

1. Lack of a modern monitoring system to follow hospitalized patients and patients who are bedridden or have particularly severe disease.

2. Lack of an internal control system to maintain communication with hospitalized patients and their families.

3. Lack of a mechanism to follow patients after discharge from the hospital and guarantee continuity of care.

4. A less than optimal medical record of patients with newly diagnosed chronic disease and of patients with existing chronic disease.

5. The absence of a social worker and a dietician in the clinic staff.

6. Insufficient health promotion and disease prevention activity on the part of the clinic staff and patients.

7. There is insufficient contact and only partial collaboration with the hospital wards, the emergency room, the hospitalization monitoring unit, and the home hospice unit.

#### Patients and environment

1. 17.5% of the clinic population are elderly, i.e., 65 years of age or older.

2. The socioeconomic status is low with 22.6% supported by the Israel Social Security Institute.

3. Low compliance rate with treatment regimen and follow-up visits.

4. The patients are unaware of other healthcare service options in the community.

5. The clinic is geographically situated close to the hospital.

### The intervention program

In light of the definition of the problem and the analysis of its causes we chose solutions that were designed to reduce the number of hospitalizations among elderly patients. The basic assumption that underpinned the choice of solutions was that improved quality of care will lead to optimization of the utilization of healthcare resources and to reduced costs. The clinic staff chose to intervene in those areas in which intervention could lead to change. For example, it would be unrealistic to intervene in factors such as the geographical location of the clinic or its socioeconomic status. However, resources could be allocated for a social worker and a dietician, and the level of cooperation with outside agencies could be enhanced. The program was developed in an ongoing manner throughout the year 2005.

#### Improvement of existing work processes and development of new processes

1. Identification of patients with multiple hospitalizations, patients with chronic diseases who were not well controlled, and patients who were hospitalized more than twice over the course of the previous year. This list of patients was disseminated among the clinic physicians who initiated appointments with them and coordinated the appointments with the clinic nursing staff. An intervention program was designed for each patient, in accordance with his or her specific needs.

2. A geriatrics specialist liaison service in the clinic:

a. The geriatrician visited the clinic once a week, reviewed the computerized patients' charts and appended electronic reminder notes regarding evaluation and treatment of elderly patients.

b. The geriatrician conducted joint meetings with physicians and patients, as a liaison.

c. Provide patients with formal internal clinic referral forms and set an appointment for them with their family physician to guarantee continuity of care for specific health problems that were identified in the joint meeting.

d. The geriatrician gave relevant lectures to the clinic staff and conducted staff meetings on issues such as prevention of falls in the elderly, polypharmacy, and the use of benzodiazepines among elderly patients.

3. Establishment of a clinic hospitalization monitor system. The team included the medical, nursing and administrative directors of the clinic. Hospital reports were printed out from the computer database and reviewed on a daily basis.

4. Health promotion and disease prevention were emphasized as critical aspects of primary care. The clinic staff prepared presentations on subjects such as the principles of disease prevention, disease prevention in adults, and early detection of colorectal and breast cancer.

#### Improvement of communication and management of patient problems

1. Individual training regarding the care of emergency cases.

2. Publication of a short newsletter on referrals to the emergency room.

3. Provision of personal telephone numbers of the medical staff to a selected group of patients (at the discretion and judgment of the treating physician).

4. The addition of a part-time social worker to help with the provision of social support to patients and the clinic staff.

5. The addition of a dietician, five hour per week, to counsel patient with metabolic and other disorders.

#### Improvement of communication with outside agencies

1. A meeting of the clinic staff with the staff of the internal medicine ward in which the clinic patients are regularly hospitalized. At the meeting the two staffs jointly discussed ways to improve hospitalization and discharge procedures as well as means to improve communication between the hospital and the clinic staff.

2. A meeting of the entire clinic staff with the emergency room staff in which the two staffs discussed issues such as improving referrals to the emergency room and improved "real-time" communication between the teams.

3. Strengthened ties with the hospital's hospitalization-monitoring unit and the development of a "service contract". The treating physician completed a structured form for every patient who remained in the hospital for more than five days. The form included the clinic physician's position as to the need for continued hospitalization and the feasibility of alternative care in the community. The clinic monitoring team transferred these recommendations to the hospital's hospitalization-monitoring team, which assessed each clinic recommendation on an individual basis. In some cases this process was also applied to patients who were hospitalized for less than five days.

4. Strengthening of ties with CHS's southern region home hospice team. Terminally ill patients who were considered suited for this type of care were provided optimal palliative care at home.

5. Direct collaboration with the person responsible for reducing patient waiting lists for outpatient consultations and elective hospitalizations.

#### Establishment in the clinic of a monitoring system to increase awareness of the importance of improved quality of care and optimal utilization of resources

1. Data on the quality indices and the use of resources were presented at staff meetings four times during the year.

2. The clinic economist held meetings with each clinic physician at which time they discussed data on the individual quality indices and use of resources in the physician's unit. These meetings were held every six months. The aim of the meetings was to apprise the physicians of what was taking place in their individual units.

3. The clinic's directorate conducted ongoing quality control assessments on quality of healthcare parameters in the clinic and the use of healthcare resources.

The medical and nursing staffs were encouraged to conduct regular visits with hospitalized and home-ridden patients and to be in touch with their families.

The clinic staff participated in the regional directorate's forum for "high-cost clinics". Reports on ongoing processes were presented at these meetings and there was an exchange of views and ideas among the staffs of the various clinics. Five forum meetings were held in 2005.

During the same time period the regional directorate initiated a program for all the clinics of the region in which all patients over the age of 65 were "mapped" and evaluated on the basis of a structured plan.

To evaluate the effectiveness of the intervention we chose the following outcomes:

1. Quality measurement outcomes such as the number of hospitalizations, the number of eye examinations for diabetic patients, and parameters of diabetes control.

2. Economic outcomes such as overall cost, cost of hospitalizations, cost of ambulatory treatments, and cost of ancillary tests.

## Results

As a result of the intervention program the actual mean overall expenditure per individual registered in the clinic decreased to $1,727 compared to $1,848 for the region, a drop of 22.5% in terms of the corresponding costs for the clinic in 2004. The adjusted hospitalization rate per 1,000 dropped by 29.3%, the adjusted number of hospitalization days per 1,000 patients dropped by 37.9%, the mean adjusted cost of hospitalization dropped by 42.8%, and the mean hospital stay dropped to 6.7 days compared to 7.1 for the region, a drop of 17.9% compared to 2004 for our clinic.

Table 2 presents details on hospitalization for clinic patients 65 years of age and older for 2005 compared to 2004, and in comparison with data from the region.

**Table 2:** Hospitalizations for patients 65 and older in 2005 (1 USD = 4.45 NIS).

	Southern district	Study clinic	Difference	Compared to region (%)	Compared to study clinic in 2004 (%)
Cost per registered patient ($)	1,849	1,727	122	--6.6	--22.5
Adjusted hospitalizations/1,000 patients	10.3	10.7	0.36	3.5	--29.3
Adjusted duration of hospitalization (days/1,000 patients)	81	82	1	1.2	--37.9
Adjusted hospitalization cost ($/1,000 patients)	19,1756	18,624	550	--2.9	--42.8
Mean duration of hospitalization (days)	7.1	6.7	--0.4	--5.5	--17.9

### Medical quality indices

Any intervention aimed at reducing costs must ensure that the quality of care is not impaired. Two hundred eighty five elderly patients received Pneumovax vaccinations in 2005 compared to 74 in 2004, an increase of 385%. Improvement was also seen in indices related to diabetes mellitus: the number of well-controlled diabetes patients (HbA_1_C < 7), the number of diabetic patients tested for microalbuminuria, and the overall follow-up.

In contrast, there was a decrease in the amount of drugs used to reduce serum lipids and in care for microalbumin. Quality-of-care data are presented in Table 3.

**Table 3:** Comparison of medical quality indices between 2004–2005 (%).

	2004	2005	Difference
Diabetics – eye examination	72.7	77.5	4.8
Diabetics – HbA_1_C measured	90.2	89.8	--0.4
Diabetics – HbA_1_C ≤ 7	41.8	54.2	12.4
Diabetics – HbA_1_C moderately elevated	44.2	37.5	--6.7
Diabetics – HbA_1_C ≥ 9	13.9	8.3	--5.6
Diabetics – LDL measured	87.4	87.2	--0.2
Diabetics – LDL ≤ 100	55.6	50.3	--5.3
Diabetics – LDL moderately elevated	27.5	31.9	4.4
Diabetics – LDL > 130	16.9	17.8	0.9
Microalbumin measured	75.4	81.3	5.9
Microalbumin treated regularly	76.5	72.0	--4.5
Medication for dyslipidemia	44.4	39.4	--5.0

### The effect of the intervention program on the overall use of healthcare resources

During 2005 there was an overall reduction of 9.9% in total clinic expenditure compared to 2004, despite an increase of 2.4% in the clinic population over the course of that year. The major cost reductions were hospitalization for acute illness (11.9%) and prolonged illness (63.0%). There was a corresponding increase in the cost of ambulatory care such as hospital day care and imaging procedures. Table 4 presents a comparison of total costs between 2004 and 2005 for all clinic age groups.

**Table 4:** Comparison of major expenses for the entire study clinic population between 2004 and 2005 in USD (1 USD = 4.45 NIS).

**Expense**	2004	2005	Difference (%)
General hospitalization	1,121,910	988,407	--133,503 (--11.9)
Elective hospitalization	495,218	476,412	--18,806 (--3.8)
Ambulatory costs	315,169	368,761	53,592 (17.0)
Imaging procedures	141,423	145,767	4,345 (3.1)
Emergency room visits	128,497	138,765	10,268 (8.0)
Hospital day care	59,157	65,028	5,871 (9.9)
Prolonged illness (nursing care)	266,785	98,817	--167,968 (--63.0)
Medications	207,759	183,321	--24,438 (--11.8)
Disposables	21,819	19,129	--2,690 (--12.3)
Different patient payments	--43,618	--39,620	3,998 (9.2)
All expenses	2,714,119	2,444,787	--269,332 (--9.9)

## Discussion

The clinic staff decided to conduct and evaluate an intervention program to reduce hospitalizations among elderly patients in the clinic because of the large effect of this parameter on costs and the utilization of the clinic resources. The program is unique in that it was developed in a structured format by the clinic staff itself. It received the support of the regional directorate as manifested in the provision of new manpower resources such as a geriatric consultant, a social worker, and a dietician. The design and development of the intervention program was an ongoing process that continued throughout the year. The focus was global rather than on a specific medical problem.

We conducted a multidimensional intervention program that included the re-engineering of existing work processes with a focus on the management of patient problems, improving communication with outside agencies and the establishment of a system to monitor healthcare parameters. Organization change in the primary care setting has been shown to be justified in previous reports [14,15] including care management for low- income seniors [16].

The success of the intervention is reflected in the reduction of the number of hospitalizations, the number of hospitalization days, the cost of hospitalization, the mean duration of hospitalization, and the overall clinic expenditure. The largest reduction in costs was for patients with prolonged hospitalizations (rehabilitation and complex nursing care). In a single clinic intervention it is possible that a small number of patients could have a significant effect on the results.

Intervention programs for the prevention of disease and the reduction of hospitalizations from the community are usually successful. An intervention program for secondary prevention of ischemic heart disease implemented by a nursing staff was shown to be cost-effective in terms of lives saved [17]. The planning of hospitalization, communication and education of patients led to earlier discharge without apparent increased morbidity in one program [18], and a similar program aimed at reducing hospitalizations for chest pain led to a reduction in hospitalization costs [19]. Support by the medical team for elderly patients in the community and cooperation with the hospital medical staff can bring about a significant improvement in hospital days, savings in expenses, and can result in better patient outcome [19,20].

Our intervention program led to an increase of 385% in Pneumovax vaccinations in the elderly. Community-acquired pneumonia is an increasingly common disease among elderly patients [21]. Early discharge from the hospital combined with support by the medical care system in the community can reduce hospital days and nosocomial infections [22]. The achievement of the intervention program may have contributed to the reduction in illness in addition to the reduction in the number of days that elderly patients spent in the hospital. Improvement could be seen in other parameters of quality that were chosen as measures of the intervention's results, for example eye examinations, HbA1c levels, and diabetes control in general (Table 3). A decrease was also seen in mortality, from 14 patients in 2004 to nine patients in 2005.

Focusing on the elderly age group in our intervention program led, in the final analysis, to a positive change in the total clinic costs. There was a significant drop in the cost of hospitalization and a corresponding increase in the use of ambulatory medical resources. These changes reflect a more optimal use of healthcare resources.

Although the development of the program and its integration into clinic work required time and effort on the part of the entire staff in the course of an intense work schedule, its implementation brought other positive developments. The entire clinic staff reported an improvement in the quality of healthcare, including improved communication with patients, families, and medical agents outside the clinic. An example of the latter is the direct communication with the regional coordinator responsible for reducing waiting times, which led to quicker treatment for the patients and improved satisfaction for all sides.

The clinic staff felt that it's work was more efficient, that relations between staff members improved, and that it was better equipped to identify and solve problems at the clinic level in an independent manner.

There are several limits to the evaluation of this intervention program. Case studies can teach a lot about processes, but their outcomes may not be readily generalizable. The number of patients in the clinic is relatively small so that a change relating to a few patients could have a significant effect on the results.

There is no way of determining the specific contribution of each of the program's elements to the final outcome, nor can we isolate the effect of specific diseases such as ischemic heart disease and pneumonia on the final results.

We decided to evaluate the intervention program in a single clinic so as to gain information about its potential benefit at the single clinic level. Other factors, e.g., a particularly severe winter in the study year or mass media reporting of bird flu, could have affected referral rates to the emergency room or even the decision as to whether to hospitalize a particular patient.

There are differences throughout the world in terms of provision of healthcare services and culture-based attitudes to healthcare. Our intervention program was designed and implemented in accordance with local needs, so we cannot be certain of their generalizability to other countries and healthcare systems. Nevertheless, we hope that our experience will aid others in meeting the challenge of improving healthcare for the elderly while attaining a more optimal allocation of healthcare resources and improving quality of care.

## Conclusion

We conclude that implementation of the intervention program in a single primary care clinic led to a reduction in hospitalizations for the elderly patient population and to a more optimal allocation of healthcare resources.

## Competing interests

The author(s) declare that they have no competing interests.

## Authors' contributions

RP initiated and coordinated the study and wrote the paper. YP was the geriatric consultant for the study and participated in writing the paper. MA headed the administration of the research project and liaison with the hospital staff. TP was a member of the project's lead team. HG served as the assistant study coordinator and held responsibility for data handling. ML was the nursing care coordinator for study and was responsibility for discharge of study patients from hospital. AB served as the external consultant for the research project and participated in writing the paper.

## Pre-publication history

The pre-publication history for this paper can be accessed here:


